# Point-of-Care Ultrasound Diagnosis of Tetralogy of Fallot Causing Cyanosis: A Case Report

**DOI:** 10.5811/cpcem.2022.8.56297

**Published:** 2022-10-22

**Authors:** Aravind Addepalli, Marco Guillen, Andrea Dreyfuss, Daniel Mantuani, Arun Nagdev, David A. Martin

**Affiliations:** * Highland Hospital-Alameda Health System, Department of Emergency Medicine, Oakland, California; † EsSalud Cusco: Hospital Nacional Adolfo Guevara Velasco, Department of Emergency Medicine, Cusco, Peru; ‡ Hennepin County Medical Center, Department of Emergency Medicine, Minneapolis, Minnesota

**Keywords:** point-of-care ultrasound, tetralogy of Fallot, emergency department

## Abstract

**Introduction:**

Tetralogy of Fallot (TOF) is a congenital heart defect with characteristic features leading to unique physical exam and ultrasound findings. In settings where there is limited prenatal screening, TOF can present with cyanosis at any time from the neonatal period to adulthood depending on the degree of obstruction of the right ventricular outflow tract.^1^

**Case Report:**

This case describes a pediatric patient who presented with undifferentiated dyspnea and cyanosis, for whom point-of-care ultrasound (POCUS) supported the diagnosis of TOF. We highlight the important role POCUS can play in a setting with limited access to formal echocardiography or consultative pediatric cardiology services.

**Conclusion:**

This report highlights the utility of POCUS as an inflection point in the diagnostic and management pathway of this patient, which is particularly important when working in a limited-resource or rural setting.

## INTRODUCTION

Congenital heart disease worldwide is reported to have a prevalence of 8–12 per 1,000 live births.^1^ Tetralogy of Fallot (TOF) is the most common cyanotic heart condition in children surviving untreated beyond the neonatal age and accounts for 7–10% of congenital heart disease globally, with a birth prevalence of 3–5 per 10,000 live births.^2^ Tetralogy of Fallot is a congenital cardiac malformation characterized by a ventricular septal defect; obstruction of the right ventricular outflow tract (RVOT); override of the ventricular septum by the aortic root; and right ventricular hypertrophy (RVH). In the United States, the diagnosis of TOF is generally made by ultrasound performed in the perinatal period; however, in settings where there is limited access to perinatal screening and formal echocardiography, clinicians rely on history, exam, and other diagnostics tests such as electrocardiogram.

Point-of-care ultrasound (POCUS) can be used to rapidly identify potential causes of dyspnea and shock in the undifferentiated patient.^3^,^4^ Cardiac POCUS, also referred to as focused cardiac ultrasound, is generally performed by non-cardiologists to ascertain only the essential information needed in critical scenarios to assist in time-sensitive decision-making.^5^ Cardiac POCUS can, therefore, be used in combination with historical and physical exam findings to recognize conditions such as TOF, particularly in settings where formal echocardiography is unavailable or impractical.^6^ Additionally, POCUS in rural areas and community hospitals has been shown to enable early diagnosis and timely initiation of medical interventions while avoiding unnecessary patient transport and associated expenditures.^7^

Tetralogy of Fallot clinically presents as cyanosis ranging from the neonatal period into adulthood depending on the degree of RVOT obstruction.^2^ Considering the variable presentation and broad differential, given clinical suspicion the emergency physician can use POCUS to evaluate for the anatomical abnormalities associated with TOF. In this report, we detail the case of a five-month-old presenting with respiratory distress and cyanosis whose care and ultimate diagnosis of TOF was driven by the POCUS findings identified during the initial resuscitation.

## CASE REPORT

A five-month-old male born via Cesarean section for twin pregnancy with no complications presented to a hospital in Cusco, Peru, with sudden onset respiratory distress. Vital signs at presentation were as follows: heart rate 150 beats per minute; blood pressure 80/47 millimeters of mercury; respiratory rate 50 breaths per minute; oxygen saturation (SpO2) 70% on room air; and temperature 36° Celsius. He was found to be in poor general condition, cyanotic and lethargic with dry mucous membranes. He was tachypneic with subcostal retractions and faint expiratory wheezing. Cardiac auscultation revealed no audible murmurs.

**Image 1:**
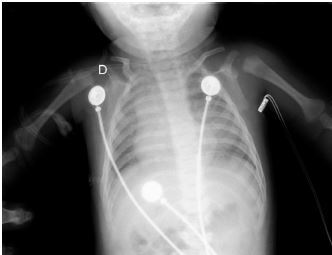
Portable anteroposterior chest radiograph showing no focal infiltrate and limited due to patient rotation.

Given these findings, the patient was suspected to be in acute respiratory failure due to severe bronchiolitis. As the patient presented early at the onset of the coronavirus disease 2019 (COVID-19) pandemic, COVID-19 remained high on the differential, given little was known regarding its effects on infants. Oxygen was administered via non-rebreather (NRB) mask with broad spectrum antibiotics and intravenous fluids (IVF) due to concern for sepsis. His labs were notable for white blood cell count of 10.6 × 10^3^ per millimeter (mm^3^) (reference range: 5×10^3^ – 10×10^3^ mm^3^) with a lymphocytic predominance; hemoglobin 20.4 grams (g) per deciliter (dL) (14–17 g/dL); creatinine of 0.4 milligrams (mg)/dL (0–0.5 mg/dL); and a lactate of 12.5 millimoles per liter (mmol/L) (0–4 mmol/L). A COVID-19 polymerase chain reaction test was negative. Chest radiograph was interpreted by the emergency physician as technically limited due to rotation with diffuse prominent interstitial markings concerning for viral pneumonia (Image 1).

The patient became more responsive after initial resuscitation with oxygen via NRB and IVF. However, he remained hypoxic with SpO2 of 76% and ongoing signs of respiratory distress. He was started on high-flow nasal cannula at nine liters per minute (L/min) with a fraction of inspired oxygen of 70% resulting in minimal improvement in overall respiratory status. Given his persistent hypoxia and cyanosis, cardiac POCUS was performed, which initially was notable for RVH raising suspicion for RVOT obstruction suggestive of a congenital heart disease.

**CPC-EM Capsule**

What do we already know about this clinical entity?

*Tetralogy of Fallot is a congenital condition with characteristic structural anomalies affecting blood flow through the heart that globally has a birth prevalence of 3–5 per 10,000 live births*.

What makes this presentation of disease reportable?

*Tetralogy of Fallot is the most common cyanotic heart condition in children surviving untreated beyond the neonatal age that can be surgically corrected once appropriately identified*.

What is the major learning point?

*Point-of-care ultrasound has an important role in identifying Tetralogy of Fallot as a cause of dyspnea in settings with limited prenatal screening and access to comprehensive echocardiography*.

How might this improve emergency medicine practice?

*With appropriate use, point-of-care ultrasound to diagnose Tetralogy of Fallot would allow crucial changes in resuscitative efforts and referral for definitive surgical treatment*.

**Image 2:**
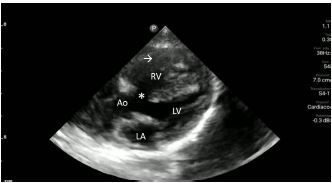
Parasternal long-axis view of the heart highlighting the findings of a ventricular septal defect (*) with an overriding aorta (Ao) and right ventricular hypertrophy (arrow). LA indicates left atrium; LV, left ventricle; RV, right ventricle.

**Image 3:**
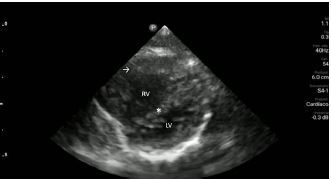
Parasternal short-axis view of the heart notable for interventricular septal flattening (*) due to right ventricular pressure overload in the setting of right ventricular hypertrophy (arrow). LV, left ventricle; RV, right ventricle.

Upon closer inspection, parasternal long-axis view revealed a ventricular septal defect with an overriding aorta and RVH concerning for TOF (Image 2). Parasternal short-axis cardiac view redemonstrated RVH with interventricular septal flattening indicative of right ventricular pressure overload from RVOT obstruction (Image 3). Pulmonary ultrasound revealed a normal A-line pattern, and POCUS assessment of the inferior vena cava showed a non-collapsing vessel.

Treatment was shifted from a focus on sepsis to TOF management. Additional IVF hydration was limited, and the patient was started on propanolol for rate control. Due to the patient’s age, the decision was made to not administer prostaglandins as he was likely not ductus dependent. He was found to improve after these interventions. Cardiology was consulted, and despite not identifying physical exam findings concerning for TOF such as skin discoloration signifying cyanosis, or a systolic thrill and ejection murmur at the left sternal border, once they were shown the POCUS images cardiology consult initiated procedures for referral to the National Institute of Cardiovascular Diseases in Lima for definitive surgical treatment. Four days later a comprehensive echocardiogram confirmed the diagnosis of TOF.

## DISCUSSION

The diagnosis of congenital heart disease in the ED can be challenging since many of the presenting symptoms can mimic other more common pathologies, as was the case in our patient whom the clinician initially suspected bronchiolitis. Thus, it is important to maintain a broad differential diagnosis, particularly when working in settings where congenital heart disease is more likely to go undiagnosed due to limited perinatal evaluation for the condition.

POCUS has been demonstrated to improve diagnostic accuracy when evaluating patients in shock.^4^,^8^ Consensus guidelines advocate for the use of cardiac POCUS by trained clinicians to help narrow the differential diagnosis and guide clinical management for both adult and pediatric patients presenting with cardiopulmonary instability.^5^ Previous case reports have similarly demonstrated the role POCUS can play in expediting the diagnosis and treatment course of children suspected of having congenital heart disease.^9^,^10^ Although cardiac POCUS is insufficient to rule it out, our case demonstrates how POCUS can be used to evaluate for gross abnormalities such as discrepancies in normal anatomy, chamber size or function, which can trigger the need for more comprehensive cardiac evaluation and formal echocardiography.

Identifying the specific anatomical abnormalities associated with congenital heart disease can pose a diagnostic challenge, particularly for clinicians who are not experienced with POCUS. Tele-ultrasound may potentially aid in cases where there is diagnostic uncertainty or limited clinical experience using POCUS. A study recently published by Médecins Sans Frontières showed that images obtained by ultrasound-naïve clinicians could be reviewed by pediatric cardiologists using a telemedicine platform to help facilitate the diagnosis and guide management of patients suspected of having congenital heart disease.^11^ Although clinicians in this study were primarily trained in image acquisition and not interpretation, this study shows promising results for the use of cardiac POCUS to help facilitate the timely diagnosis of congenital heart disease in limited-resource settings where access to pediatric consultative services or comprehensive echocardiography would be otherwise impractical. More broadly speaking, our case also underscores the important role that POCUS has in the ED management of infants presenting with undifferentiated dyspnea or shock in both high- and low-resource settings.

## CONCLUSION

POCUS is an invaluable tool for evaluating the undifferentiated patient presenting with dyspnea, particularly when working in a limited-resource setting where there is reduced access to timely laboratory and diagnostic studies. This case highlights the important role of POCUS, particularly in a setting with limited prenatal screening, for congenital cardiac abnormalities and limited access to comprehensive echocardiography or pediatric cardiology consultation services. Here, the POCUS findings of a ventricular septal deficit with an overriding aorta and RVH in the hands of a trained clinician suggested TOF and changed the course of the initial resuscitative efforts leading to ultimate referral to a tertiary care center for definitive surgical treatment.

## Supplementary Information

Video 1 Parasternal long-axis view of the heart. Ventricular septal defect (*); overriding aorta (Ao); right ventricular hypertrophy (arrow); left atrium (LA); left ventricle (LV) right ventricle (RV).

Video 2 Interventricular septal flattening due to right ventricular pressure overload (*); right ventricular hypertrophy (arrow); left ventricle (LV) right ventricle (RV).
